# Prognostic significance of VEGF expression in patients with bulky cervical carcinoma undergoing neoadjuvant chemotherapy

**DOI:** 10.1186/1471-2407-8-295

**Published:** 2008-10-11

**Authors:** Chel Hun Choi, Sang Yong Song, Jung-Joo Choi, Young Ae Park, Heeseok Kang, Tae-Joong Kim, Jeong-Won Lee, Byoung-Gie Kim, Je-Ho Lee, Duk-Soo Bae

**Affiliations:** 1 Department of Obstetrics and Gynecology, Samsung Medical Center, Sungkyunkwan University School of Medicine, Seoul, Korea; 2 Department of Pathology, Samsung Medical Center, Sungkyunkwan University School of Medicine, Seoul, Korea

## Abstract

**Background:**

The prediction of response to treatment would be valuable for managing cervical carcinoma with neoadjuvant chemotherapy.

**Methods:**

To this end, the expression of VEGF was analyzed by immunohistochemistry using paraffin-embedded pre-treatment cervical biopsy tissues. This study included 29 patients with bulky IB to IIA cervical squamous cell carcinoma treated with neoadjuvant chemotherapy.

**Results:**

Fifteen (51.7%) of 29 patients were scored as VEGF-positive. Response to chemotherapy (complete response or residual tumor with less than 3 mm stromal invasion) was observed in eight patients (27.6%), and it was negatively associated with VEGF expression (*P *= 0.009). With logistic regression analysis, VEGF positivity continued to be an independent predictor for poor response (*P *= 0.032). In addition, the progression-free survival rate was significantly lower in patients with VEGF-positive tumors (*P *= 0.033).

**Conclusion:**

Pretreatment assessment of VEGF expression may provide additional information for identification of patients with cervical cancer who had a low likelihood of response to neoadjuvant chemotherapy and an unfavorable prognosis.

## Background

Carcinoma of the uterine cervix is the second most common cancer in women, but the prognosis remains very poor in bulky or locally advanced disease [1]. Although concurrent chemoradiation (CCRT) is now considered standard treatment, neoadjuvant chemotherapy (NAC) has been adopted to improve the prognosis for these cases [2,3]. The development of convenient and reliable biomarkers predicting the treatment response would be valuable for patient management. If non-responsive tumors could be identified before NAC, using predictive biological factors, these patients could be allocated to CCRT. Furthermore, it would be reinforced if the biological factors found do not affect the response to CCRT.

The correlation of angiogenesis with either metastasis or a poor prognosis has been reported in various cancers [4-6]. Among the angiogenic factors, vascular endothelial growth factor (VEGF) has been shown to have a pivotal role in tumor angiogenesis. However, the correlation between VEGF expression and prognosis in patient with cervical cancer has been inconsistent; this may be because of the marked heterogeneity of patient disease stages and treatment modalities in reported studies [7-9]. Although, there are some reports that show that VEGF plays an important role in patient response to chemotherapeutic agents, [10] there is little information available on its predictive value for treatment response in patients receiving NAC for cervical carcinoma. Therefore, we evaluated whether VEGF may have predictive value for patient response to NAC in cases with bulky cervical carcinoma.

The aim of the present study was to investigate the expression of VEGF and their possible role as predictors of response to NAC in patients with bulky cervical carcinoma.

## Methods

### Patients and samples

Of the patients with locally advanced cervical carcinoma, who had presented to the Samsung Medical Center, 46 patients with stage IB2 to IIB enrolled into a phase II trial of NAC [11]. Among them, 29 patients with stage IB2 to IIA and squamous cell histology were selected to minimize heterogeneity of the patient population studied (15 patients with stage IIB and 2 patients with adenocarcinoma were excluded). The Institutional Review Board at Samsung Medical Center (Seoul, Korea) approved the protocol, and all patients provided written informed consent before entry into the trial. None of the patients was pretreated with any other chemotherapy or radiotherapy before the NAC. The median patient age was 47 years (range, 33 to 70). Twenty (69.0%) patients had stage IB2 disease and nine (31.0%) patients had stage IIA. The other clinicopathologic characteristics are shown in Table 1.

**Table 1:** Immunoreactivity of VEGF according to clinicopathologic characteristics of the cervical carcinoma patients

	No. of patients	VEGF expression		
			
Variables		Positive	Negative	*P*
Total	29	15	14	
Age				
≥50 years	12	7	5	0.55
< 50 years	17	8	9	
Stage				
IB2	20	9	11	0.28
IIA	9	6	3	
Cervical tumor size				
≥5 cm	14	9	5	0.19
< 5 cm	15	6	9	
Clinical node involvement				
Yes	11	7	4	0.32
No	18	8	10	
SCC Ag level				
≥5 ng/ml	14	8	6	0.57
< 5 ng/ml	15	7	8	

^a^(n = 29)

^a^The *P*-value was determined using Chi-square test or Fisher's exact test.

### Treatment and response

Cisplatin-based chemotherapy (combination of vincristine 1 mg/m^2^, mitomycin-C 10 mg/m^2 ^and cisplatin 75 mg/m^2^) was administered every 3 weeks [11]. A type III radical hysterectomy with pelvic and paraaortic lymph node dissection was performed within 3 weeks of the administration of the third cycle of NAC in all patients. Following radical surgery, adjuvant radiotherapy was performed if lymph node metastasis, parametrial involvement or a positive resection margin were found.

In this study, the tumor response was evaluated pathologically. Complete response (CR) was defined as a complete disappearance of the invasive tumor in the cervix with negative nodes, and optimal pathologic response (OPR) was defined as a residual tumor with less than 3 mm stromal invasion. The 3-mm threshold used was chosen because it represents the maximal extension of FIGO stage IA1 cervical tumor, which is usually considered cured after local resection. And the role of OPR as a possible surrogate endpoint for survival in the neoadjuvant setting, has been reported [12]. In the present study, patients with CR or OPR were grouped together as responders.

### Immunohistochemistry and evaluation

Paraffin-embedded tissue blocks of formalin-fixed cervical biopsy specimens taken pre-treatment, were processed for conventional histological assessment by hematoxylin and eosin (H&E) staining and immunohistochemical (IHC) analysis using the avidin – biotin – peroxidase method. VEGF protein expression was detected by mouse anti-human monoclonal VEGF (ab1316) antibody (Abcam, Inc., Cambridge, UK), using conventional peroxidase methods [13]. In brief, 4 μm thick sections were deparaffinized in xylene, dehydrated through graded alcohol concentrations and incubated in citrate buffer (pH = 6.0) for 5 min using a household microwave oven at 800 W. After microwave exposure, the slides were allowed to cool to room temperature. The slides were briefly washed with PBS and incubated for 15 min with 3% hydrogen peroxide in methanol to block endogenous peroxidase activity. The antibody to VEGF was diluted 1:100 and incubated for 1 h at room temperature. Biotinylated antimouse/rabbit antibodies (DAKO) at a dilution of 1:500 were used as the second antibody. Negative controls included substitution of the monoclonal antibody with normal mouse IgG of the same concentration as the monoclonal antibody [see Additional file 1]. Sections of corpus luteum were used as positive control for VEGF immunostaining. After washing, ABC (DAKO) was applied and diaminobenzydine was used for visualization. Tissue sections were lightly counterstained with hematoxylin and then examined by light microscopy.

Assessment of the staining was scored independently by two investigators (SYS and CHC) without knowledge of the clinicopathological findings. Expression was defined as positive if distinct staining of the cytoplasm was observed in at least 10% of tumor cells [14]. The scoring by the two investigators was similar. In the cases of disagreement, slides were reevaluated and discussed until consensus was achieved.

### Statistical analysis

Fisher's exact probability test or the Chi-square test was used to analyze frequency data. Multiple logistic regression models were used to identify independent prognostic factors for patient response. The tumor stage, nodal involvement, tumor size, and SCC-Ag levels were entered into the logistic regression models. Disease-free survival was measured by the Kaplan-Meier method. Differences between groups were tested using the log-rank test. To determine the independent prognostic value for patient disease-free survival, a Cox regression model was constructed using tumor stage, LN involvement, tumor size, SCC-Ag level, and IHC status as covariates. A *P*-value of less than 0.05 was considered significant. SPSS 10.0 (SPSS Inc., Chicago, IL) was used for the statistical analysis.

## Results

**Figure 1:**
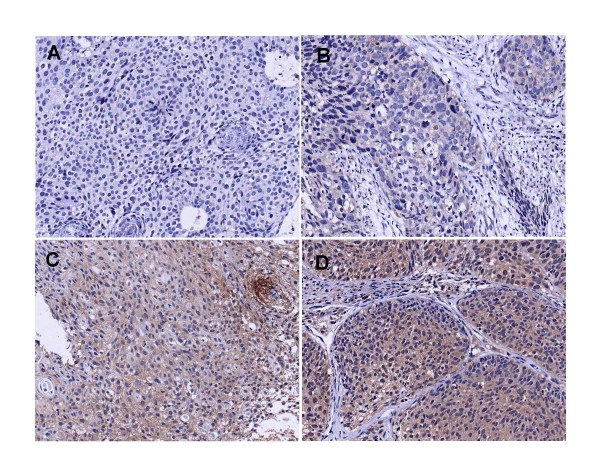
Representative examples of VEGF staining in bulky cervical carcinoma showing cases with no staining (A), weak staining (B), moderate staining (C) and strong staining (D) (×200).

Figure 1 show representative results of IHC staining. Fifteen (51.7%) of 29 patients were scored as VEGF-positive (Table 1). Table 1 lists the positivity of the proteins according to clinicopathologic characteristics.

Pathologic responses, including complete and optimal responses, were observed in eight (27.6%) patients, with no response in the remaining 21 (72.4%) patients (Table 2). VEGF expression was shown to be highly associated with tumor susceptibility to NAC. The response rate of VEGF-positive tumors was significantly lower than VEGF-negative tumors (7% *vs. *50%, *P *= 0.009). When logistic regression was applied, VEGF positivity continued to be an independent predictor of poor response to treatment (*P *= 0.032).

**Table 2:** Clinicopathologic parameters and the expression of VEGF as predictors of response to neoadjuvant chemotherapy in patients with bulky cervical carcinoma

			Pathologic responder		Nonresponder		
						
Characteristics	No. of patients	RR	CR (n = 4)	OPR ^a ^(n = 4)	(n = 21)	*P*^b^	*P*^c^
Age, years						0.16	
Median			58	47	46		
Range			38 – 60	34 – 60	33 – 70		
Cervical tumor size (cm)						0.07	0.22
Median			3.8	3.5	5.0		
Range			3.2 – 6.0	3.0 – 5.6	3.7 – 7.1		
Clinical node involvement						0.98	0.22
Yes	11	27.3%	2	1	8		
No	18	27.8%	2	3	13		
Stage						0.67	0.54
IB2	20	30.0%	2	4	14		
IIA	9	22.2%	2	0	7		
SCC Ag level						0.47	0.36
≥5 ng/ml	14	21.4%	1	2	11		
< 5 ng/ml	15	33.3%	3	2	10		
VEGF expression						0.009	0.032
Negative	14	50.0%	3	4	7		
Positive	15	6.7%	1	0	14		

^a^Residual tumor but only with less than 3 mm stromal invasion

^b^Rank sum test or Chi-square test/Fisher's exact test

^c^Logistic regression analysis with stage, tumor size, SCC-Ag level, and lymph node involvement as a covariate.

RR, pathologic response rate; CR, complete response; OPR, optimal pathologic response

**Figure 2:**
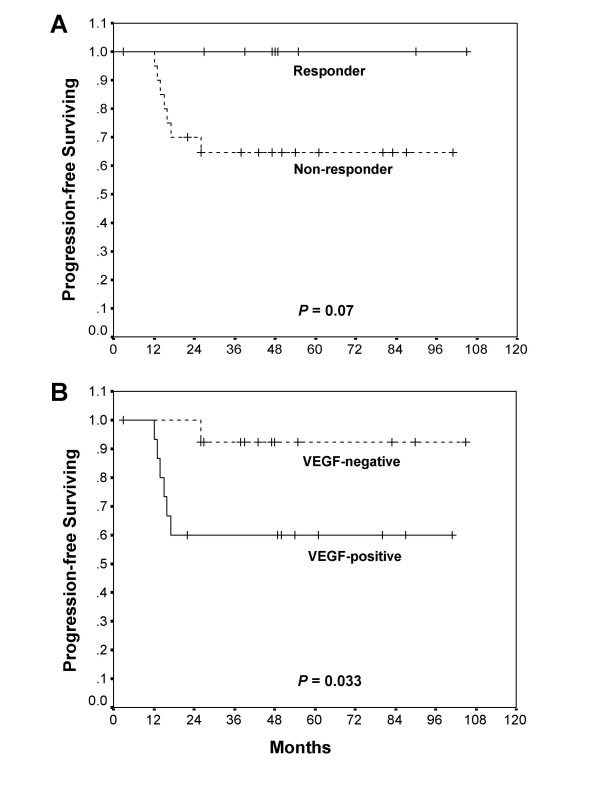
**Disease-free survival curve as a function of pathologic response (A) and immunoreactivity to VEGF (B).** The *P*-values were determined using the log-rank test.

With a median follow-up period of 48 months (range, 3 to 105), three (10.3%) of 29 patients died of disease and recurrence occurred in seven (24.1%). The overall 5-year disease-free survival rate was higher in the responder group than the non-responder group (100% *vs. *65%), although this difference was not statistically significant (*P *= 0.07) (Figure 2A). The progression-free survival rate was significantly lower in patients with VEGF-positive tumors (*vs. *VEGF-negative tumors, *P *= 0.033) (Table 3 and Figure 2B). VEGF-positivity was identified as an independent predictor of patient disease-free survival using a Cox regression model (*P *= 0.037; hazards ratio, 11.4; 95% CI, 1.15 – 112.58) (Table 3).

**Table 3:** Univariate and multivariate analysis of clinicopathologic factors affecting disease-free survival rate

			Univariate analysis	Multivariate analysis	
				
Characteristics	No. of patients	5-y DFS (%)	*P*	Relative risk (95% CI)	*P*
Cervical tumor size					
≥5 cm	14	69.2	0.50	0.66	0.53^a^
< 5 cm	15	80.0		(0.18 – 2.42)	
Clinical node involvement					
Yes	11	70.6	0.49	2.30	0.39
No	18	81.8		(0.35 – 15.10)	
Stage					
IB2	20	73.3	0.83	0.67	0.64
IIA	9	77.8		(0.13 – 3.58)	
SCC Ag level					
≥5 ng/ml	14	69.2	0.50	1.03	0.45^a^
< 5 ng/ml	15	80.0		(0.95 – 1.12)	
VEGF expression					
Negative	14	92.3	0.033	11.40	0.037
Positive	15	60.0		(1.15 – 112.58)	

^a ^Treated as a continuous variable.

## Discussion

In this study, the clinical significance of IHC-positivity for VEGF in pre-treatment biopsy specimens was examined in 29 patients with cervical squamous cell carcinoma undergoing NAC. The most interesting finding of our study was the strong correlation between VEGF expression and response to NAC. These results suggest that patients with cervical cancer who are positive for VEGF expression are less likely to benefit from NAC. Therefore, patient monitoring for VEGF expression may provide an important determinant for the differential treatment of bulky cervical cancer. Although it is very difficult to develop the best alternative strategy in such VEGF-positive patients, concurrent chemoradiation or radical surgery without delay or the addition of anti-VEGF therapy may be useful in improving the prognosis of those patients [15]. However, additional study is needed for confirmation of these findings.

The reason for the correlation between VEGF positivity and chemoresistance is unclear. The presence of increased vascularity may suggest improved tumor oxygenation and drug delivery; this may improve response to chemotherapy. However, this remains contradictory in many cases [16,17]. Unlike normal blood vessels, tumor vessels are structurally and functionally abnormal, *i.e.*, the formation of tortuous and saccular blood vessels that are poorly organized and hyperpermeable [18]. These abnormalities can increase resistance to blood flow and impair blood supply, and therefore compromise the delivery and effectiveness of conventional cytotoxic therapies [19]. It has also been suggested that the role of angiogenic factors in the treatment response may be governed by hypoxia. Piret and colleagues suggested that HIF-1α (key proteins regulating angiogenesis) has both pro- and anti-apoptotic effects [20]. Mild hypoxia causes the expression of various anti-apoptotic proteins, whereas severe hypoxia leads to cell death, at least in part, through stabilization of p53 by HIF-1α [20]. The overall balance of the activation effects of HIF-1α may depend on the type of cancer and treatment modality used [21].

Clinical studies on the correlation between VEGF expression and prognosis have reported inconsistent results [7-9,22]. Some reasons for the conflicting results include the following. First, the patient groups studied were heterogeneous in terms of disease stages and treatment modalities. Second, the studies were prone to sampling errors because the neovascularization status of the tumor could not be reliably determined with a single measurement of only a small portion of the whole tumor. Jain reported that not only does the microcirculation vary from one tumor to the next, but within the same tumor, it varies both spatially and temporally [23].

## Conclusion

The present study showed that the assessment of VEGF expression in pretreatment biopsy specimens could provide additional information to identify patients with a poor chance of response to NAC and unfavorable prognosis in patients with bulky cervical carcinoma.

## Competing interests

The authors declare that they have no competing interests.

## Authors' contributions

CHC and SYS collected the data, performed analysis and prepared the manuscript. JJC, YAP, HK and TJK performed the immunoassays and statistical analysis. JWL, BGK and JHL selected the cases and interpreted the results. DSB designed the study concept, interpreted the results and approved the final manuscript.

## Pre-publication history

The pre-publication history for this paper can be accessed here:



## Supplementary Material

Additional file 1 **Negative controls for VEGF staining.** All slides show negative staining (×200). Click here for file
