# Supplementary figures and images for: Prognostic significance of VEGF expression in patients with bulky cervical carcinoma undergoing neoadjuvant chemotherapy
Source: BMC Cancer. 2008 Oct 11;8:295. doi: 10.1186/1471-2407-8-295 (PMC2572070; doi:10.1186/1471-2407-8-295)

## Slide 1
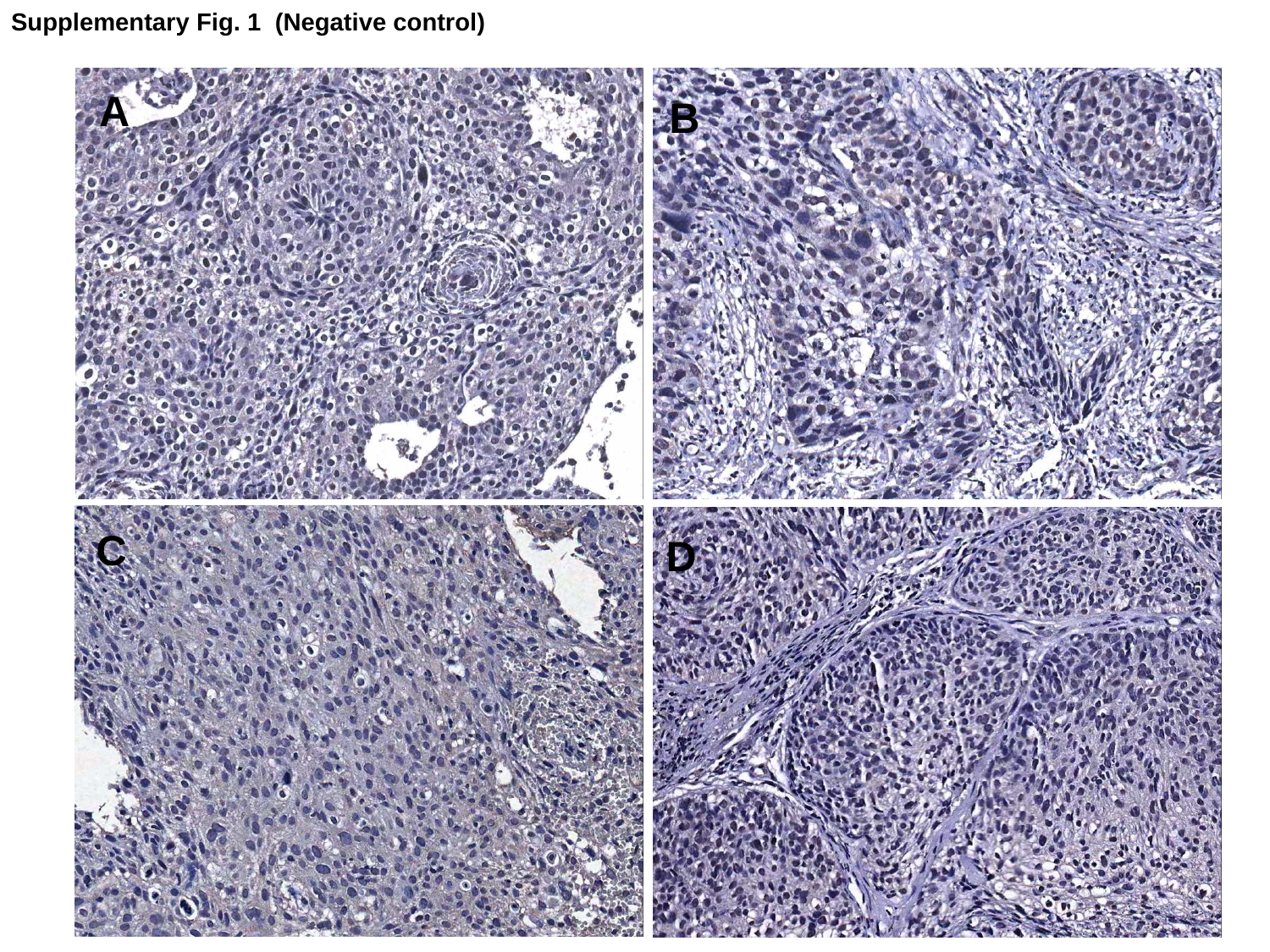

Supplementary Fig. 1 (Negative control)
A
B
C
D

Supplement: Additional file 1 — Negative controls for VEGF staining. All slides show negative staining (×200). [file 1471-2407-8-295-S1.ppt]
